# Optimization of the incubation parameters for biogenic synthesis of WO_3_ nanoparticles using Taguchi method

**DOI:** 10.1016/j.heliyon.2022.e10640

**Published:** 2022-09-15

**Authors:** Dali Vilma Francis, T. Aiswarya, Trupti Gokhale

**Affiliations:** a Department of Biotechnology, BITS Pilani Dubai Campus, P. O. Box 345055, Dubai International Academic City, Dubai, United Arab Emirates; b Department of Physics, BITS Pilani Dubai Campus, P. O. Box 345055, Dubai International Academic City, Dubai, United Arab Emirates

**Keywords:** Tungsten nanoparticles, Green synthesis, Taguchi method, Regression analysis, ANOVA, *S. maltophilia*

## Abstract

Green synthesis of metal nanoparticles is gathering attention due to eco-friendly processing. Tungsten oxide (WO_3_) nanoparticles have immense applications as semiconductors, antimicrobials and photo thermal materials but their synthesis using biological systems is hitherto unpublicized. The paper discusses synthesis of WO_3_ nanoparticles using *Stenotrophomonas maltophilia* and the optimization of physico-chemical parameters of incubation which influence the growth and metabolism of the bacterium and consequently the size of the WO_3_ nanoparticles. The biogenic synthesis of WO_3_ nanoparticles was confirmed by ATR-FTIR and X-ray diffraction analysis. Taguchi and analysis of variance method was applied to optimize the physico-chemical parameters (pH, temperature, time, aeration rate and concentration), considering particle size and poly dispersity index (PDI) of the nanoparticles as the experimental responses. Under the design of experiments technique, Taguchi's L27 array was selected to determine the optimal process parameters which could significantly reduce the particle size and PDI of WO_3_ nanoparticles. Statistical analysis by signal-to-noise ratio, regression analysis and ANOVA (95% confidence level) on experimental responses confirmed pH and aeration as most influential while temperature and time as least influential parameters. pH 8, Temperature 40 °C, aeration 200 RPM, time 3 days and concentration of sodium tungstate at 1 mM (p3t3r3d3c1) was the most effective level and parameters combination for smallest particle size and PDI of WO_3_ nanoparticles. Regression models developed for particle size and PDI exhibited a linear regression of 97.80% and 90.89% respectively, while the confirmation test validated the size and PDI of the experimental values against predicted results. SEM image of WO_3_ nanoparticles illustrated the same particle size as that predicted, further validating the model. The study can be applied to optimize any process parameters in the industry or on biological systems.

## 1 Introduction

Nanotechnology is a rapidly growing field of science with numerous applications benefiting the society. Nanoparticles have been applied in various fields such as agriculture (Francis et al., 2020), antimicrobial polymer composites (Francis et al., 2022), drug delivery (Mitchell et al., 2021), electronics (Pandey, 2022) etc. Nanoparticles possess certain unique characteristics such as optical, electrical, catalytic etc. which are different from their bulk counterparts due to their increased surface area to volume ratio (Francis et al., 2020; Naz et al., 2020; Salata 2004). The global metal nanoparticle market was valued at US$ 25,373.92 million in 2020 and is predicted to increase at a compound annual growth rate (CAGR) of 15.9% from 2021 to 2028, to reach US$ 81,567.38 million (Inshakova et al., 2020; Market Research; Nanoparticles, 2021), due to the increased demands by its end users such as pharma, healthcare, cosmetics, agriculture (Francis et al., 2020), food packaging industry (Francis et al., 2022), drug delivery (Mitchell et al., 2021), electronics (Pandey, 2022) etc.

The size of nanoparticles and their mode of synthesis is very crucial for its application. Smaller the size of the nanoparticles, more diverse are its application. Poly dispersity index (PDI) which is a measure of size heterogeneity of the nanoparticles in a solution is a critical factor considered during the synthesis of nanoparticles. The PDI value ranges from 0 to 1 with 0 indicating a monodispersed solution while higher values indicate a wide size heterogeneity of the particles in solution (Kumar et al., 2012; Orellano et al., 2017). Metal nanoparticles can be synthesized using physical, chemical, or biological methods. Physical methods use the top-down approach which involves application of large amounts of energy which results in raising the environmental temperature and requires a long time for achieving the thermal stability (Iravani et al., 2014). Chemical approaches, including chemical reduction of bulk metal salts using a variety of organic and inorganic reducing agents and solvents (Iravani et al., 2014). The use of toxic chemicals for their synthesis limits the application of such nanoparticles in medicine and healthcare (Modan and Plăiașu, 2020). Hence the green synthesis of nanoparticles using biological systems ranging from simple prokaryotic bacterial cells to eukaryotic fungi and plants has been grabbing global attention (Begum et al., 2022; Mohanpuria et al., 2008). Nanoparticle synthesis using bacteria has been demonstrated for gold, silver, zinc, cadmium, iron, and magnetite; using yeasts and fungi for silver, cadmium, lead, and gold; algae for silver and gold, while plants for palladium, zinc oxide, platinum, and magnetite besides silver and gold (Debaditya and Rajinder, 2005). Biological methods require consideration of critical aspects, such as type of organism, genetic properties of organisms, optimal conditions for cell growth and enzyme activity as well as optimal reaction conditions (Iravani et al., 2014). Sizes and morphologies of the nanoparticles can be controlled in biological methods by altering some physico-chemcial parameters such as substrate concentration, pH, temperature, electron donor, biomass of the organism, mixing speed, and exposure time (Iravani et al., 2014). Hence if not standardized, biological systems particularly bacterial systems exhibit a major drawback regarding the non-uniformity of the nanoparticle size and shape as the process involves the use of biomolecules such as enzymes for the synthesis. This has raised a great scientific interest in understanding the mechanism for nanoparticle growth and the influencing conditions. Controlling the physicochemical parameters for the growth and metabolism of the bacteria could influence the seeding of nanoparticles (Castro et al., 2014, Park et al., 2010). Hence, identifying the influencing parameters contributing to the formation of desired shape, particle size and PDI of nanoparticles are important for developing the process for nanoparticle synthesis (Hasnain et al., 2019; Shi et al., 2011). It is therefore important to design experiments to study the interactions between the influencing factors that assist in reducing the particle size and PDI (Draheim et al., 2015).

Statistical experimental designs provide an easier and efficient approach to optimize functional variables. Taguchi method is one of the robust designs which is widely used in the field of engineering, to determine the optimum process parameters (Adnani et al., 2010; Karna & Sahai, 2012; Vankanti and Ganta 2014; Pouretedal et al., 2017, Pundir et al., 2018; Robinson et al., 2004). Safaei et al., 2019 have applied the Taguchi method for three variables at three levels using 9 experimental runs for evaluating the optimal conditions for synthesis of cellulose MgO nano-composite with strongest antibacterial activity. The Taguchi approach, which is based on orthogonal array (OA) for Design of experiment (DOE), involves the investigation of a process by a series of independent parameters at different levels (Karna & Sahai, 2012; Morali et al., 2018). Using this method, it is possible to optimize the process conditions with reduced number of experiments while considering the implications of individual factors which organize the relationship between variables and operating conditions to eventually determine the optimal levels of performance (Shafiee et al., 2019). On the contrary, traditional design of experiment is a less preferred method as it focuses on studying a single variable at a time, resulting in large number of experimental combinations which make the optimization laborious and expensive (Medan et al., 2017). Also, traditional DOE fails to identify the most influential parameter as well as the impact of interaction between the process parameters on the process and the products developed (Weissman and Anderson, 2015). Though the Taguchi method exhibits several advantages, its applications in optimization of biological processes is less. A few researchers have explored the use of Taguchi method for optimizing the synthesis of bio-products such as enzymes, ethanol, metabolites from biological systems (Dasu et al., 2003; Rao et al., 2008). The process optimization provides reliable results by suggesting major changes in the operations, manufacturing, and quality control for numerous industries (Rao et al., 2008).

However, due to the complexities involved while working with a living system, there have been no reports on the optimization of the particle size and PDI of nanoparticles synthesized using bacterial systems. In the present study Taguchi L27 orthogonal array, signal to noise ratio and ANOVA were employed to analyse the effect of various incubation parameters for smallest particle size and PDI of nanoparticle, following which regression analysis was performed to derive the regression predictive models which were also compared with the Taguchi prediction (Manivel and Gandhinathan, 2016).

Tungsten oxide nanoparticles have emerged as one of the most sort after metal nanoparticle due to its application as novel antimicrobial agent (Duan et al., 2019) and as active photothermal nanomaterial due to their ability to absorb light in the near-infrared (NIR) region and its effective conversion to heat (Wu et al., 2019). WO_3_ nanoparticles also have immense applications (Yao et al., 2021) as semi-conductors (Diez-Cabanes et al., 2021), gas sensors (Dai et al., 2021), memory devices (Ji et al., 2016), etc. A soil bacterium, *Stenotrophomonas maltophilia* isolated from the desert soil of UAE was found to exhibit multi metal resistance (unpublished reports) including resistance to sodium tungstate. Advanced studies on *S. maltophilia* have attributed this high resistance to its ability of reducing toxic tungsten ions to nontoxic or less toxic WO_3_ nanoparticles. Present study is the first report on bacterial synthesis of WO_3_ nanoparticles and optimization and modelling of incubation parameters for *S. maltophilia.* The present investigation aims to understand the impact of incubation parameters and to select the optimal incubation conditions using Taguchi method for reduced and uniform sized WO_3_ nanoparticles.

## 2 Materials and methods

### 2.1 Materials

Luria Bertani broth was procured from Himedia Laboratories Pvt. Ltd, India and Sodium tungstate was of analytical grade, purchased from Sigma-Aldrich.

### 2.2 Methods

#### 2.2.1 Growth of Stenotrophomonas maltophilia

Overnight grown culture of *Stenotrophomonas maltophilia* was inoculated in 100 ml fresh sterile Luria Bertani broth (pH7) adjusting to 0.8 OD at 600 nm. The bacterial culture was incubated at 30 °C for 24 h at 150rmp.

#### 2.2.2 Preparation of WO_3_ nanoparticle

The grown culture of *S. maltophilia* was further incubated with sodium tungstate and subjected to physicochemical variation to study the effect of different parameters on the particle size and PDI of the synthesized nanoparticles. After incubation, the cells were ruptured by sonication using QSONICA sonicator and the cell debris was removed by centrifugation. The supernatant was filtered through 0.22 μm cellulose acetate membrane and dialyzed using a snakeskin dialysis membrane (10 K MWCO) for 24 h against deionized water (Milli Q systems Merck). The dialyzed samples were subjected to particle size analysis in Malvern Zeta sizer Nano DS and the samples were then lyophilized in a Buchi, lyovapor L-200 and considered for further characterization.

#### 2.2.3 Characterization of nanoparticles

The dialyzed samples were subjected to Dynamic light scattering (DLS) to measure the particle size using Nano ZS series Malvern Zetasizer. The lyophilized samples of the nanoparticles were imaged under JEOL JSM-7600F FEG-SEM to study the morphology and size of the individual nanoparticles. The lyophilized samples of the nanoparticles were also analysed for X-Ray Diffraction on a Bruker AXS Kappa APEX II CCD X-ray diffractometer operated at 40 kV and 40 mA with Cu Kα radiation (1.54 Å) as a source. A continuous scan mode was applied with a step width of 0.020°, sampling time of 57.3 s and measurement temperature of 25 °C. The scanning range of 2θ was between 3° and 80°. FTIR analysis was performed using ATR-FTIR Shimadzu IRSpirit, to confirm the presence of capping agents in microbial synthesized nanoparticle.

#### 2.2.4 Design of experiment

Bacterial synthesis of WO_3_ nanoparticles is mediated by enzymes and metabolic state of the bacterium. The physico-chemical parameters of incubation greatly influence bacterial growth and the metabolic state (Nevot et al., 2008; Patra and Baek, 2014), thereby affecting the size of the synthesized nanoparticles. Several studies have demonstrated the reduction in size of metal nanoparticles due to increased incubation temperature (Fayaz et al., 2009), pH (Salazar-Bryam et al., 2021), aeration (Castro et al., 2020), and salt concentration (Mishra et al.,.2011; Honary et al., 2015). The authors were curious to learn the effect of incubation time on the size of the metal nanoparticle as there were no report on this parameter. It is hence important to optimize the incubation parameters for bacterial growth and synthesis of WO_3_ nanoparticles. The five most influential physico-chemical parameters (factors) of incubation are aeration rate during incubation (RPM), temperature (°C) of incubation, concentration (mM) of tungsten salt, time (day) of incubation and pH of the growth media (pH) and hence, were selected in the current study at three different levels based on preliminary unpublished results. The selected incubation parameters and their levels are given in Table 1. A full factorial design would require 243 runs in triplicate (ie 729 runs) to identify the optimal combination of the selected incubation parameters. The Taguchi orthogonal array (OA) design however delivers a more simplified way to frame the combination of experimental parameters compared to the factorial method and reduces the number of experimental runs to 27, thereby lowering the experimental cost and time (Rekab and Shaikh, 2005; Shi et al., 2020). Since we have considered 5 incubation parameters (factors) at three different levels, Taguchi L27 orthogonal array (OA) (five factors with 3 levels) design was selected to determine the optimal parameters for the response variables, particles size and PDI. The experimental plan for 27 runs is described in Table 2. Taguchi design explains three types of optimization criteria for the response variables, (i) Larger the better, (ii) Nominal the better, (iii) Smaller the better (Mia, 2018). Nanoparticles of smaller size and lower PDI demonstrate wider applications and hence in the current study, criteria iii, smaller the better was chosen to compute the signal to noise (SN) ratio to analyse the influence of control parameters on dependent variable.

**Table 1:** Incubation parameters and their levels selected for the optimization of biogenic synthesis of WO_3_ nanoparticles by *S maltophilia*.

Incubation Parameters		Units	Levels		
			1	2	3
pH	p	pH	6	7	8
Temperature	t	°C	25	30	40
Aeration	r	RPM	0	100	200
Time	d	day	1	2	3
Concentration	c	mM	1	3	5

**Table 2:** Experimental design using Taguchi L27 orthogonal array design (OA) and the average values of particle size, PDI and SN ratio for each factor level combination designed.

Experimental Runs	Levels of Incubation Parameters					Particle size			PDI		
	p	T	r	d	c	S/N ratio	Size (nm)	Std dev	S/N ratio	PDI	Std dev
1	1	1	1	1	1	−44.92	176.27	4.70	4.32	0.61	0.06
2	1	1	1	1	2	−45.50	188.30	5.93	3.66	0.66	0.04
3	1	1	1	1	3	−46.17	203.39	5.48	3.21	0.69	0.04
4	1	2	2	2	1	−42.04	126.42	3.12	7.64	0.41	0.02
5	1	2	2	2	2	−42.32	130.56	4.01	7.06	0.44	0.00
6	1	2	2	2	3	−43.28	145.88	4.51	5.02	0.56	0.02
7	1	3	3	3	1	−41.20	114.77	6.20	10.41	0.30	0.01
8	1	3	3	3	2	−41.83	123.50	4.25	9.53	0.33	0.01
9	1	3	3	3	3	−42.06	126.77	5.70	7.62	0.42	0.02
10	2	1	2	3	1	−41.00	112.18	6.55	9.39	0.34	0.02
11	2	1	2	3	2	−41.83	123.39	2.62	8.08	0.39	0.01
12	2	1	2	3	3	−42.45	132.66	5.86	7.66	0.41	0.04
13	2	2	3	1	1	−41.08	113.26	8.58	10.45	0.30	0.02
14	2	2	3	1	2	−41.49	118.65	3.56	9.70	0.33	0.02
15	2	2	3	1	3	−41.93	124.94	4.38	9.34	0.34	0.01
16	2	3	1	2	1	−42.71	136.57	4.49	7.26	0.43	0.02
17	2	3	1	2	2	−42.92	139.95	7.35	6.87	0.45	0.03
18	2	3	1	2	3	−44.08	159.99	10.45	6.73	0.46	0.03
19	3	1	3	2	1	−37.92	78.72	7.98	13.13	0.22	0.02
20	3	1	3	2	2	−38.76	86.75	9.32	10.45	0.30	0.03
21	3	1	3	2	3	−39.84	98.21	8.10	8.74	0.37	0.06
22	3	2	2	3	1	−40.39	104.63	10.31	8.00	0.40	0.01
23	3	2	2	3	2	−40.69	108.23	4.75	6.91	0.45	0.01
24	3	2	2	3	3	−41.94	125.08	8.67	6.86	0.45	0.02
25	3	3	3	1	1	−38.97	88.87	6.73	11.85	0.26	0.03
26	3	3	3	1	2	−39.34	92.71	6.24	10.76	0.29	0.01
27	3	3	3	1	3	−40.30	103.53	11.25	10.58	0.30	0.05

The SN ratio was calculated for each factor level combination using Eq. (1) (Manivel and Gandhinathan, 2016).

(1) $$SN=-10\text{​}*\log\left(\Sigma \left(Y^{2}\right)/n\right)$$

*Y* = responses for the given factor level combination, *n* = number of responses in the factor level combination

The confirmatory tests were performed to compare the difference between predicted and experimental values.

#### 2.2.5 Analysis of variance

ANOVA was performed with the Taguchi OA to determine the most and least influential incubation parameters for the synthesis of tungsten nanoparticles (Reddy et al., 2015).

#### 2.2.6 Regression model development

Regression is a statistical modelling process that establishes relation between the response (particle size or PDI) and input parameters (selected incubation parameters). Regression can be modelled as linear or polynomial relationships (George et al., 2005, Rekab and Shaikh, 2005). In the current investigation, linear regression analysis (Minitab 19.0) was used to create predictive mathematical models for the dependent variables of size and PDI as a function of pH, temperature, aeration, salt content, and time, respectively.. The predicted values from Taguchi OA (Minitab 19.0 software) and linear regression equation and experimental values were compared and were validated by SEM analysis. Potential of the developed models were evaluated by R^2^ (coefficient of determination) values (Sivaiah and Chakradhar, 2019).

## 3 Result and discussion

**Figure 1:**
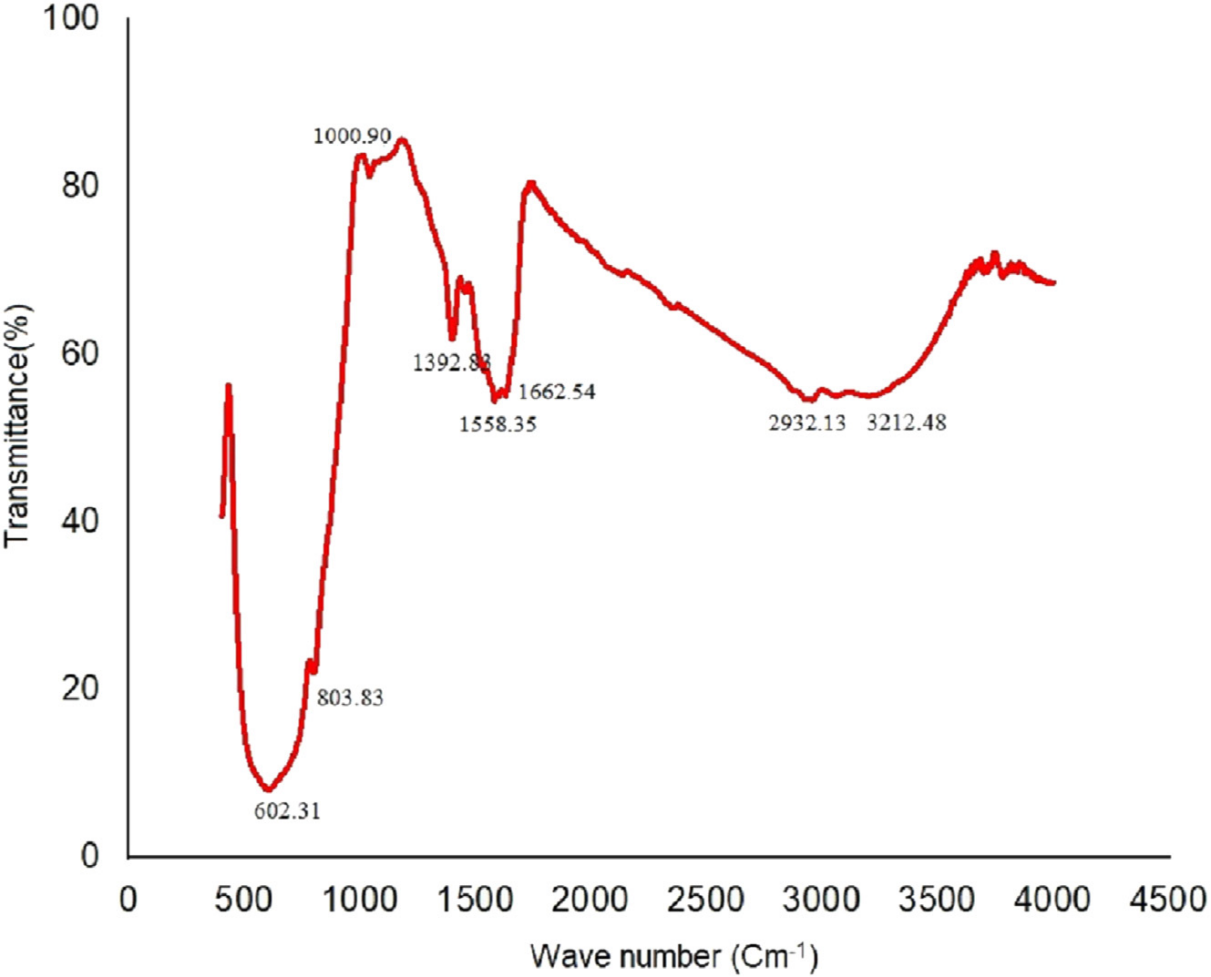
FTIR spectra of WO_3_ nanoparticles synthesized by *S maltophilia* using the optimized incubation conditions predicted by Taguchi method. The analysis was performed in the mid-IR range on ATR-FTIR Shimadzu IRSpirit.

**Figure 2:**
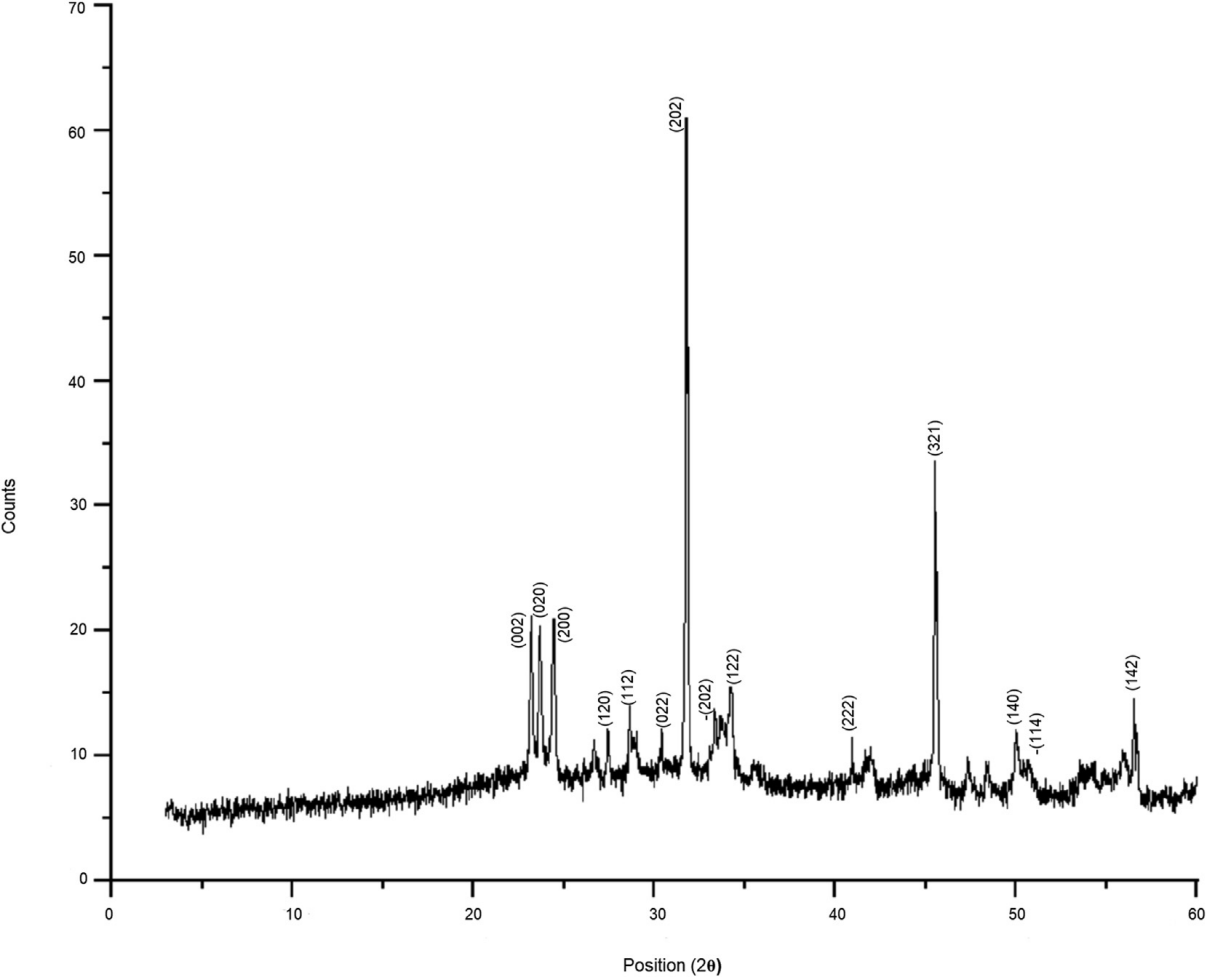
XRD analysis of WO_3_ nanoparticles synthesized by *S maltophilia* using the optimized incubation conditions predicted by Taguchi method. The analysis was executed on Bruker AXS Kappa APEX II CCD X-ray diffractometer operated at 40 kV and 40 mA with Cu Kα radiation (1.54 Å) as a source. The XRD patterns obtained were compared with the standard JPCDS card No: 83-0950.

The biogenic synthesis of WO_3_ nanoparticles by *Stentrophomonas maltophilia* was confirmed by the ATR-FTIR and XRD analysis (Figures 1 and 2).

ATR-FTIR analysis (Figure 1) of the lyophilized nanoparticles exhibited a few prominent absorption peaks at 1662 cm^−1^, 1558 cm^−1^, 1392 cm^−1^, 1000 cm^−1^, 1073 cm^−1^, 803 cm^−1^ and 602 cm^−1^. The peaks at 602 cm^−1^ and 803 cm^−1^ were imputed to the O–W–O stretching and W–O–W bending vibrations, while the peak at 1000 cm^−1^ is attributed to W= O stretching, thereby suggesting the biosynthesis of WO_3_ nanoparticle (Najafi-Ashtiani et al., 2018).

The IR absorption at 1662 cm^−1^ corresponds to C=O stretching, while 1558 cm^−1^ are unique absorption peaks associated with the C=O stretching of amide I and C–N stretching and N–H bending vibrations of amide II in the peptides respectively. Absorption at 1073 cm^−1^ reflects the C–N stretching vibration for aliphatic amines while 1392 cm^−1^ is due to the symmetric and asymmetric CH_3_ bending vibrations from amino acids (Miller et al., 2013). The presence of water adsorbed to the nanoparticle is distinctly evident from the broad spectrum around 3200 cm^−1^ (Bodade et al., 2017). These absorption peaks for the amides signify the presence of a protein cap around the WO_3_ nanoparticles, commonly observed during biogenic synthesis and are strikingly absent in chemically synthesized nanoparticles.

XRD pattern of the lyophilized nanoparticles displayed a reflection index as (002), (020), (200), (202), (222), (321), (140), and −(114). The XRD patterns (Figure 2) on comparison with standards (JPCDS card No: 83-0950) established the presence of WO_3_ nanoparticles in the lyophilized powder (Ghasemi and Jafari, 2017).

The combined effect of different incubation parameters, viz; pH, temperature, aeration, concentration, and time on the size and PDI of biogenic formation of WO_3_ nanoparticles by *Stentrophomonas maltophilia* were established with Taguchi OA L27 method using Minitab-19 software. Regression analysis of the data points examined the collaborative effect of the parameters and predicted the best combination of parameters and their levels for size and PDI.

The experimental runs according to Taguchi orthogonal array (L27) were performed and the response values for mean particle size and PDI are listed in Table 2. All experimental trials were performed in triplicates to countenance the accuracy of the response of Taguchi orthogonal array.

The results for each run exhibited a wide range of particle sizes and PDI. The smallest size of WO_3_ nanoparticles was obtained in experimental run 19 (*p3t1r3d2c1*) with particle size 78.72nm and PDI 0.22, while experimental run 3 (*p1t1r1d1c3*) resulted in larger particle size at 203.39nm as well as higher PDI 0.69 (Table 2). Thus, it is evident that the combination of the physico-chemcial parameters for incubation influence the size and PDI of the biosynthesized WO_3_ nanoparticles. Similar influences have been previously reported during synthesis of silver, gold, ferric chloride and tellurium, nanoparticles (Fayaz et al., 2009; Salazar-Bryam et al., 2021; Castro et al., 2020; Mishra et al., 2011; Honary et al., 2015).

## 4 ANOVA for particle size and PDI

ANOVA was performed to understand the influence of the incubation parameters and determine the contribution towards the particle size and PDI of tungsten nanoparticles (Kharisma et al., 2017; Kowalczyk, 2014). The ANOVA output for particle size and PDI of the tungsten nanoparticles are expressed in Table 3 pH was observed to be the most influential parameter for the smallest size of the synthesized nanoparticles with 55.25% contribution, followed by aeration (32.13%), concentration of tungsten salt (7.79%), duration of incubation (2.69%), least by temperature (1.39%). Similarly, the most significant contributor towards the smallest PDI of the nanoparticles was rate of aeration at 47.26%, followed by pH (31.62%), temperature of incubation (8.84%), concentration of tungsten salt (8.21%), and least significant being duration of incubation (1.08%). Thus, pH and rate of aeration were the most significant incubation parameters for smallest particle size and homogeneity of the WO_3_ nanoparticles. The effect of varying pH on size of biogenically synthesized gold nanoparticles has been previously reported by Konishi et al. (2007). The report has demonstrated a significant increase in nanoparticle size from 10–20 m to 15–200 nm with a decrease in pH from 7.0 to 2.8. Similar reports have been published by He et al. (2007) and Deplanche and Macaskie (2008) for the biogenic synthesis of gold nanoparticles. Rate of aeration not only enhances mass and oxygen transfer but also ensures homogenous conditions in the nutrient medium by mixing. The agitation caused due to mixing also assists in dispersing the nanoparticle aggregates thereby ensuring a monodispersed nanoparticle suspension (Kim et al., 2003; Mantzouridou et al., 2002; Castro et al., 2020; Rispoli et al., 2010).

**Table 3:** ANOVA output on contribution of the incubation parameters on particle size and PDI of tungsten nanoparticles (at 95% confidence level).

Particle size							PDI					
Source	DF	SS	MS	%	F	*p*	DF	SS	MS	%	F	*p*
pH	2	56.3106	28.1553	55.25	590.72	0.000	2	0.113316	0.056658	31.62	54.09	0.000
Temperature	2	1.4182	0.7091	1.39	14.88	0.000	2	0.-31678	0.015839	8.84	12.36	0.001
RPM	2	32.7401	16.3701	32.13	343.45	0.000	2	0.16938	0.08469	47.26	84.99	0.000
Time	2	2.745	1.3725	2.69	28.80	0.000	2	0.003853	0.001927	1.08	0.16	0.855
Concentration	2	7.9378	3.9689	7.79	83.27	0.000	2	0.029429	0.014715	8.21	18.08	0.000
Error	16	0.7626	0.477	0.75			16	0.010717	0.00067	2.99		
Total	26	19995.7		100.00			26	0.358374		100.00		

DF-degree of freedom, SS- sum of squares, %-percentage contribution, F–F value, *p*-p value.

It is highly commendable to obtain a low error percentage (0.75% and 2.99% for particle size and PDI respectively) considering the biological synthesis of tungsten nanoparticles using bacteria, *S. maltophilia*, thereby reflecting on the consistency and reproducibility of the results (Table 3). Higher the percentage contribution of an incubation parameter, greater is its influence on synthesis of smaller sized particles with lowered PDI. This thus explains the importance of each incubation parameter on the particle size and PDI of the biosynthesized WO_3_ nanoparticles. The results of ANOVA were in accordance with those obtained using the Taguchi OA on basis of the SN ratio (Table 4).

**Table 4:** Mean SN ratio by factor level for incubation parameters on particle size and PDI.

Process Parameters	Mean S/N ratio on particle size					Mean S/N ratio on PDI				
	Level 1	Level 2	Level 3	Delta (Δ)	Rank	Level 1	Level 2	Level 3	Delta (Δ)	Rank
pH	−43.26	−42.17	−39.80	**3.46**	1	8.497	8.386	9.699	3.201	2
Temperature	−42.04	−41.66	−41.49	0.55	5	7.627	7.885	9.070	1.443	4
RPM	−43.26	−41.28	−40.68	2.58	2	5.981	8.671	9.930	**3.949**	1
Time	−42.19	−41.54	−41.49	0.7	4	8.208	8.101	8.273	0.172	5
Concentration	−41.14	−41.63	−42.45	1.31	3	9.162	8.113	7.307	1.855	3

### 4.1 Effect of process parameters

Understanding the optimal level of each influencing parameter is essential for the overall process optimization. The SN value was used to determine the most significant parameter/s and most contributing level towards smallest particle size and lowest PDI. The SN ratio by factor level for incubation parameters (p, t, r, d, c) on particle size and PDI of WO_3_ nanoparticle synthesized by *S. maltophilia* are presented in Table 4. The delta (Δ) value was calculated by determining the difference between highest SN and lowest SN for each parameter. The larger delta (Δ) values for a parameter signify a greater influence of the parameter on the incubation process and correspond to a smaller variance which results in better performance of the experiment (John et al., 2013; Külekcı 2013). Considering our criteria as smaller-the-better for the particle size, pH of the incubation medium showed the larger delta (Δ) value compared to other incubation parameters. Larger delta signifies a large variation in the particle size as an effect of the pH change (Konishi et al., 2007; He et al., 2007; Deplanche and Macaskie, 2008). Based on the Δ value we can hence conclude, pH of the incubation medium is the most influential parameter in the bacterial synthesis of WO_3_ nanoparticles followed by aeration of the medium, concentration of the tungsten salt, duration of incubation and lastly temperature of incubation. Surprisingly the most influential parameter for the lowest PDI of the synthesized WO_3_ nanoparticles was not in-line with the parameters influencing the particle size. The rate of aeration (RPM) of the incubation medium (Table 4) had the most influence on the PDI. Aeration of the medium was performed by shaking the flask at varying RMP on the orbital shakers. Higher RMP helps in dispersing the nanoparticle aggregates thereby resulting in a monodispersed nanoparticle solution (Kim et al., 2003; Mantzouridou et al., 2002; Castro et al., 2020; Rispoli et al., 2010). Followed by rate of aeration are pH of the incubation medium, temperature of incubation and concentration of the tungsten salt in the respective order of influence. The least influencing parameter for PDI was the duration of incubation, as it exhibited the lowest delta value. The influence of the parameters towards the lowest PDI of the nanoparticles was observed to be in accordance with the results obtained using ANOVA.

### 4.2 Selection of optimum condition for particle size and PDI

**Figure 3:**
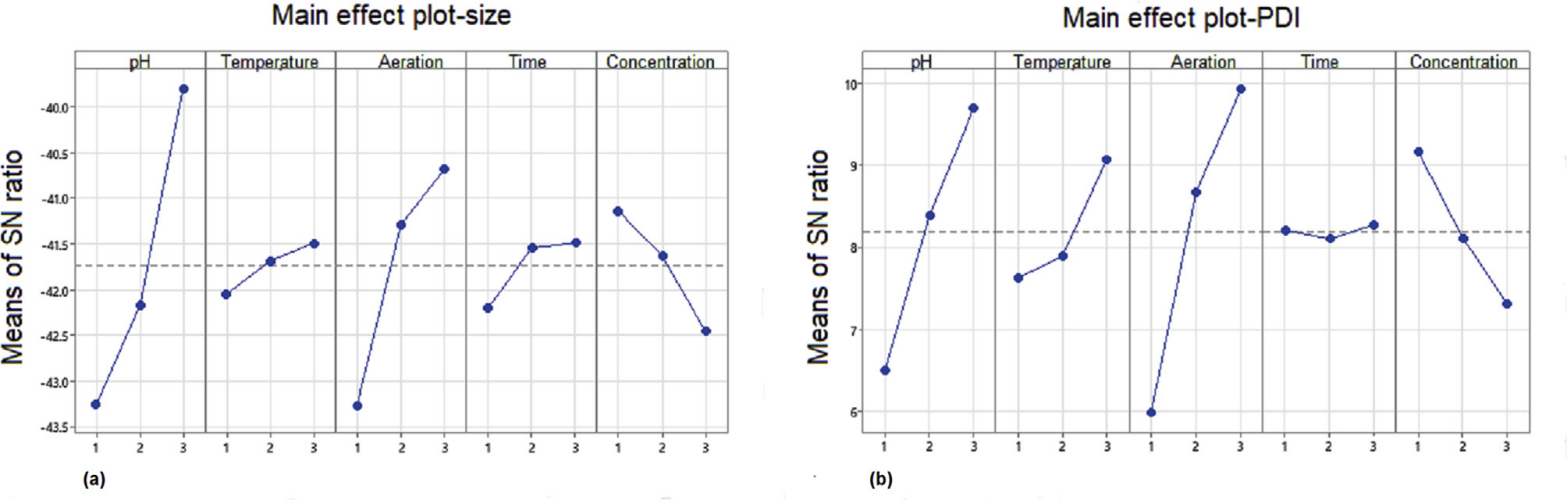
Main effect plot of SN ratio for particle size (a) and PDI (b) of WO3 nanoparticles synthesized by *S maltophilia* grown in Luria Bertani media.

Having understood the contribution and influence of each incubation parameter through the variation in SN ratio, the most favourable levels of each parameter were decided from the Main effect plot of SN ratio for particle size and PDI (Figure 3) using Minitab 19.0 software. A strong influence of the incubation parameter levels was observed on the particle size of the synthesized WO_3_ nanoparticles as depicted in Figure 3(a) pH of the incubation media was observed to be the most influencing parameter. Altering the pH tends to influence the surface zeta potential of the particles such that an increased zeta potential reduces the flocculation and aggregation of nanoparticles (Marsalek, 2014; Sousa and Teixeira, 2013). The effect of alkaline pH on zeta potential of ZnO nanoparticles was studied by Marsalek (2014). The study reported a significant alternation in the isoelectric point of the ZnO nanoparticles due to increased alkalinity thereby influencing the aggregation of nanoparticles resulting in increased particle size. Similarly, lowering the pH of the medium also resulted in increased particle size (Konishi et al., 2007; He et al., 2007; Deplanche and Macaskie, 2008). Hence, in the current finding, near neutral range was selected for fine tuning the optimization. The study demonstrated reduced particle size at pH8 which is most favourable during bacterial synthesis of nanoparticles and hencethe smallest sized particles with lowest PDI were achieved at pH 8. Following pH, the rate of aeration (RPM) was the next most influential parameter for particle size. A higher aeration rate of 200 RPM resulted in smaller particles size, while maintaining the WO_3_ salt concentration at 1mM. Higher aeration rate prevents the aggregation of nanoparticles (Kim et al., 2003; Mantzouridou et al., 2002; Castro et al., 2020; Rispoli et al., 2010) and thus resulted in lowering the particle size and PDI of the nanoparticles. Similarly, increased temperature at 40 °C (Fayaz et al., 2009; Maliszewska, 2011) and time of incubation for 3 days lowered the particle size. Fayaz et al. (2009) demonstrated the effect of temperature on the size of biogenically synthesized silver nanoparticles by *Trichoderma viride*, while Lade and Shanware (2020) demonstrated that increased incubation temperature resulted in rapid reduction of Ag + ions followed by nucleation of silver nuclei, resulting in the development of small size silver nanoparticles. During biogenic synthesis of nanoparticles, application of maximum possible temperature is recommended for optimal growth and the enhanced enzymatic activity for nanoparticle synthesis (Gurunathan et al., 2009). Jameel et al., (2020) reported the fact that reaction temperature affects the size of platinum nanoparticles and controls the rate of formation of nanoparticles i.e. at higher reaction temperature yields faster rate of particle growth (Jameel et al., 2020). There are two distinct effects reported on the influence of incubation time on size of biogenically synthesized nanoparticles. (Gericke and Pinches, 2006) reported, if the reaction mixture is incubated for longer time than the optimum, the nanoparticles tend to aggregate causing increased particle size, while some nanoparticles may even shrink upon longer storage (Darroudi et al., 2011; Baer, 2011). The later seems to be the case with the WO_3_ nanoparticles synthesized by *S. maltophilia.*

Surprisingly, increased concentration of the tungsten salt is observed to exhibit a negative effect on the desired size and PDI of nanoparticles, probably due to enhanced nucleation resulting in formation of aggregates. Concentration of the tungsten salt has a relatively greater influence on the particle size and PDI, comparable to that exhibited by temperature of incubation, while time of incubation was the least influential.

From Figure 3(a), (b) the predicted optimum parameters for smallest particle size and PDI are *p3t3r3d3c*, ie; pH 8 (*p3*), incubation temperature at 40 °C (*t3*), aeration of the medium at 200 RPM (*r3*), duration of incubation for 3 days (*d3*) and concentration of tungsten salt at 1 mM (*c1*).

### 4.3 Mathematical modelling

In the current study, linear regression analysis was performed using Minitab 19.0 software to develop the predictive mathematical models for dependent variables namely, particle size and PDI as function of pH of medium, temperature of incubation, rate of aeration, duration of incubation and concentration of tungsten salt. The predictive equations obtained from the regression analysis are shown in Eqs. (2) and (3) respectively for particle size and PDI.

(2) $$Mean\;Particle\;Size=1196.5-119.55\text{ ​}p-21.36t+0.0872r-264.0d\;+1.12c+0.4254t^{2}+3.87d^{2}+0.490c^{2}+43.87p*d\;-2.075\text{ ​}t*d-0.00863\text{ ​}r*c-8.99\text{ ​}r^{2}+2.067d^{2}\;+11.92t*d$$

(*R*^2^ = 95.5%)

(3) Mean ​PDI=2.012−0.2370p−0.000947r−0.6899d+0.01686c+0.09348p∗d+0.000341r∗d

(*R*^2^ = 97.01%)

From the Regression equations, the predicted values for particle size and PDI can be calculated for each trial run. Similarly, the equations can also be used to predict the outcome of any parameter change on the dependent variables (Asadi et al., 2014, Mandal et al., 2011). The predicted values for particle size and PDI for some experimental runs are reported in Table 5.

**Table 5:** Predicted values and confirmation test results by Taguchi method and regression equation for random runs and optimized combination.

Run	Size					PDI				
		Taguchi		Regression			Taguchi		Regression	
	Actual	Prediction value	Error%	Prediction value	Error%	Actual	Prediction value	Error%	Prediction value	Error%
1	176.27	167.79	5.05	174.74	−1.611	0.608	0.611	−0.47	0.529	2.63
7	114.77	105.02	9.28	107.27	−0.406	0.302	0.309	−2.50	0.291	3.69
15	124.93	120.24	3.91	122.59	−1.462	0.341	0.363	−5.97	0.365	−6.53
21	98.20	91.41	7.43	96.23	2.572	0.365	0.335	8.98	0.315	16.13
25	88.87	80.30	10.67	92.43	4.454	0.255	0.239	6.74	0.259	−1.56
Optimized parameters (*p3t3r3d3c1*)	70.32	76.115	−7.61	63.290	11.108	0.353	0.361	−2.12	0.371	−4.76

Another method for predicting the dependent variables is by developing the equation by Taguchi method. Hence, the Taguchi predicted values of multiple SN ratio at optimal factor level ε0 was also calculated using Eq. (4)

(4) $$\varepsilon 0=\varepsilon m+\overset{j}{\underset{i=1}{\sum }}\left(\varepsilon i-\varepsilon m\right)$$

ε0- prediction, εm- Total mean SN ratio, εi-Mean SN ratio at optimal level, j- No: of input process parameters.

The predicted values for particle size and PDI by application of Eq. (4) are presented in Table 5 for some experimental runs. Several researchers have used the Taguchi method for optimization of the non-biological synthesis of nanoparticles, but there are no reports on its use for developing a mathematical model for predicting the dependent variables (Do Kim et al., 2007: Kim et al., 2009 and Chiang et al., 2011). El-Moslamy et al. (2017) have used the Taguchi OA for optimizing the synthesis of silver nanoparticles from *T. harzianum*, but also have not developed a mathematical model for predicting the size or influence of independent variables on the dependent variables.

**Figure 4:**
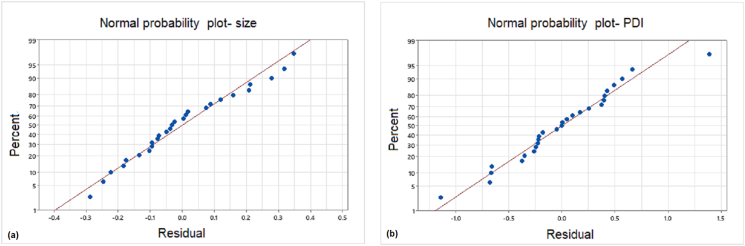
Normal probability plot developed for (a) particle size (b) PDI exhibiting R^2^ values as 95.5% and 97.01% respectively.

The capability of both models was checked by using the coefficient of determination (R^2^). The regression models for particle size and PDI have high R^2^ values as 95.5% and 97.01% respectively, which is commendable considering the use of bacterial systems for the synthesis of WO_3_ nanoparticles. Residual plots (Figure 4(a) and (b)) generated to check the significance of coefficients for the predicted models were obtained as straight-line indicating the normal distribution of the residual errors in the model, thus reassuring the significance of the developed coefficient models.

#### 4.3.1 Confirmation test

Validating the proposed design of an experiment is vital to prove the anticipated improvement on the process response by the optimal parameters proposed by the matrix test. Five runs from the L27 Taguchi OA (runs 1, 7, 15, 20 & 26) were randomly selected along with a run using the optimized levels of incubation parameters (***p3t3r3d3c1*)** for validating the regression and Taguchi models. Each experimental run was performed in triplicates and the average particle size and PDI was compared with the predicted values from Taguchi (eq. 4) and regression (Eqs. (2) and (3)) equation. Table 5 depicts the actual experimental values and predicted values for particle size and PDI by Taguchi method (eq. 4) and regression analysis (Eqs. (2) and (3)). The results from both regression and Taguchi model evince a significant accordance (p value >0.05) between the experimental values and the predicted values, with a very insignificant error. The error values obtained are <11% for both particle size and PDI, confirming the reliability and reproducibility of the data. This implicates the application of the mathematical modelling in predicting the optimal levels of the incubation parameters for the synthesis of WO_3_ nanoparticles. Similar models can be developed to optimize incubation parameters for synthesis of other biological products such as polysaccharides, proteins, enzymes, vitamins, acids, or industrially important metabolites (Fürtauer et al., 2018: Malhotra and Chapadgaonkar, 2020). The models can also contribute towards predicting the influential of process conditions for any chemical, or industrially important process. Thus, the mathematical model developed may find applications in refining any process parameter with less hassle.

**Figure 5:**
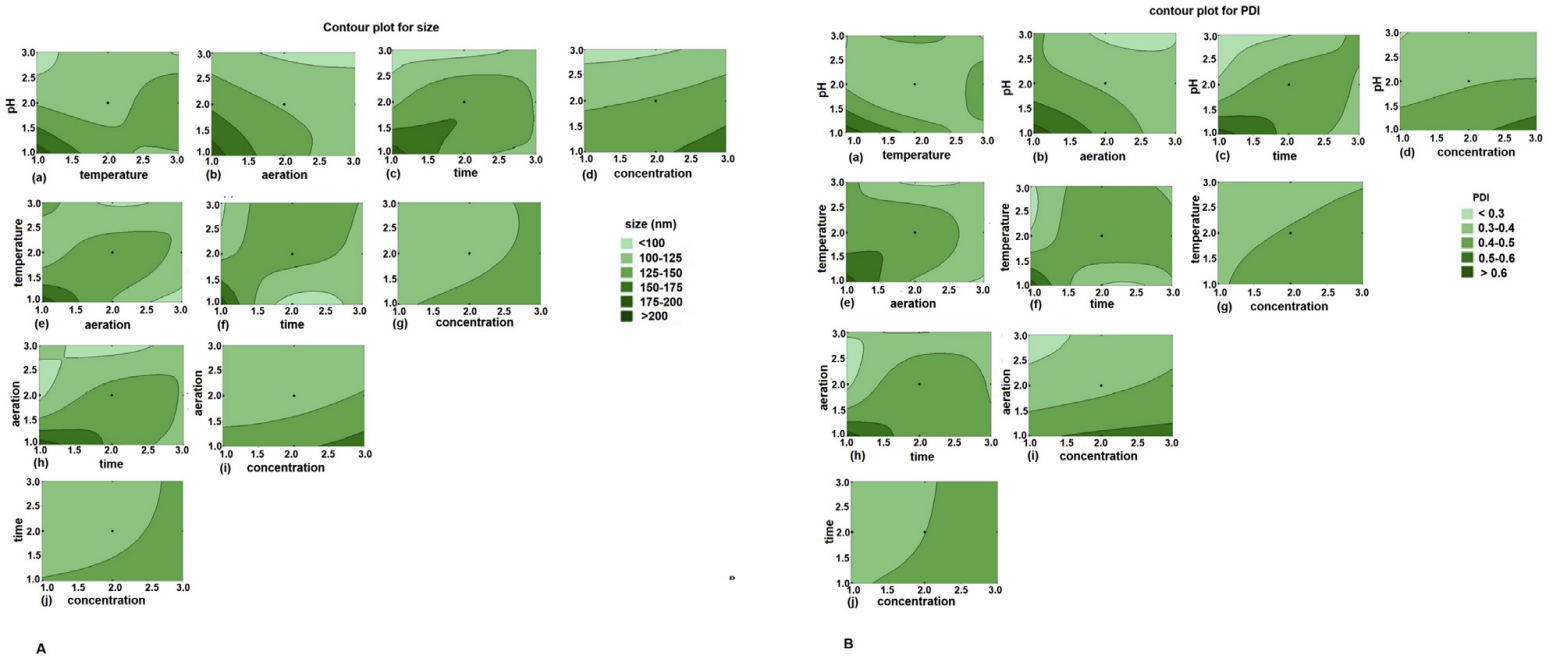
2-Dimensional Contour plots illustrate the influence of different incubation parameters on particle size (A) and PDI (B). The different panels demonstrate the combined influence of 2 parameters on particle size and PDI. (a) pH and temperature, (b) pH and aeration, (c) pH and time, (d) pH and concentration, (e) temperature and aeration, (f) temperature and time, (g) temperature and concentration, (h) aeration and time, (i) aeration and concentration, (j) time and concentration.

Contour plots assist in projecting the effects of each independent variable and the interaction between these variables on the dependent variable response (Yang et al., 2014). The contour plots for particle size and PDI as a function of incubation parameters are constructed by fixing two of the independent variables (Figure 5). This gives a diagrammatic representation of relationship between the experimental responses and input variables. The plots assess the relation among the input parameters (*p,t,r,d,c*) and two response variables (particle size and PDI) by examining distinct contours of the predicted response variables. Figure 5A and 5B depict the contour plots describing the influence of the process parameters on particle size and PDI respectively. The contour plots clearly exhibit a positive relationship with pH and aeration, a rise in pH and aeration leads to formation of smaller sized particle with a lower PDI (Figure 5A(b) and 5B(b)). Interestingly, similar trend is observed for the interaction between temperature and aeration for particle size and PDI. Figure 5A(h) and 5B(h) illustrates that reduced size and PDI can be achieved with higher RPM and number of days. Figure 5A(i) and 5B(i) depict that higher RPM and lower concentration leads to the generation of smaller sized nanoparticle with low PDI. Panels a, c, d in Figure 5A and 5B suggest reduced particle size and PDI can be obtained by combining high pH with high/low temperature, high aeration, less days, and lower concentration ***(p3t1r3d1c1****)* This combination of the incubation parameters is different than that predicted by Taguchi SN ratio and ANOVA (***p3t3r3d3c1)***. However, on analysing the SN ratio for temperature and time (days) the difference is insignificant with p value 0.952 and 0.939 respectively at 95% CI.

**Figure 6:**
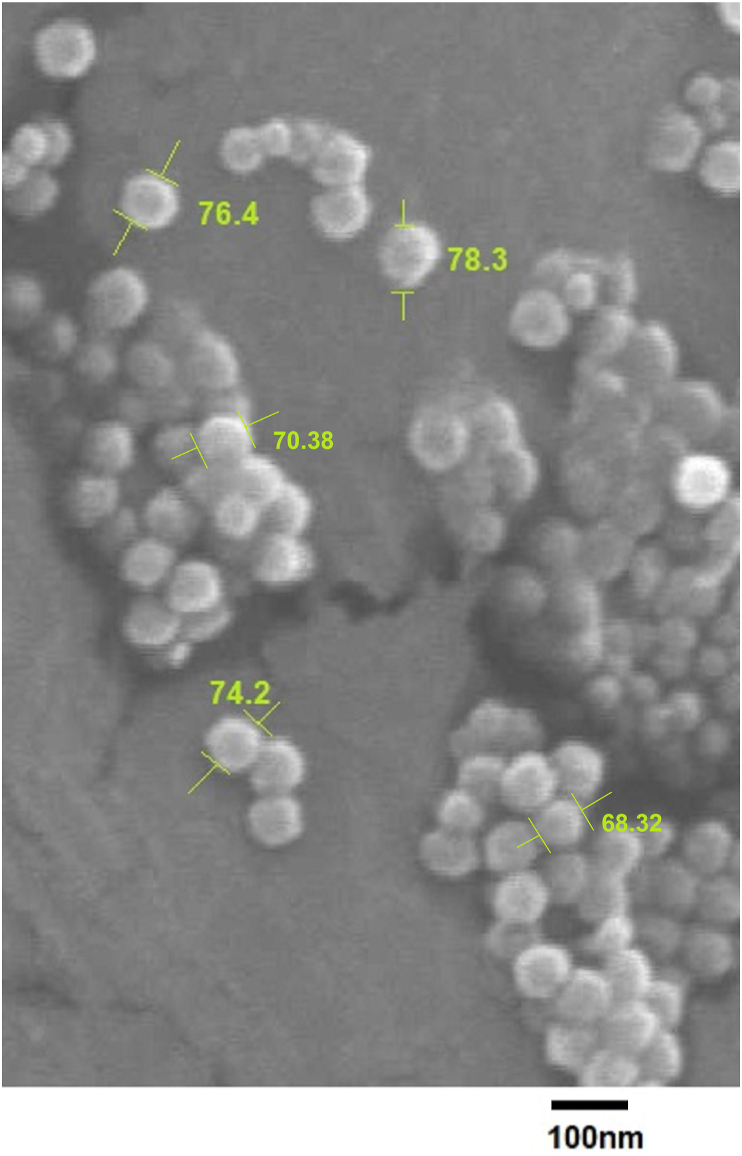
SEM image of WO_3_ nanoparticles synthesized by *S maltophilia* using the optimized incubation conditions predicted by Taguchi method. The image was captured on JEOL JSM-7600F FEG-SEM at 3.0kV.

SEM image (Figure 6) of the lyophilized WO_3_ nanoparticles synthesized by *Stenotrophomonas maltophilia* using ***p3t3r3d3c1*** incubation parameters displays the actual particle size. All WO_3_ nanoparticles appear spherical in shape and are in 70–80 nm range. The physically measured size of the nanoparticles through SEM image agrees with the predicted values by Taguchi and regression analysis. FTIR (Figure 1) and XRD (Figure 2) analysis also confirm the biosynthesis synthesis of tungsten nanoparticles using ***p3t3r3d3c1***combination of incubation parameters.

## 5 Conclusion

Tungsten nanoparticles have a plethora of applications in diverse fields and hence there is a great interest on their synthesis. The current processes employed for their synthesis either use physical or chemical methods which are either polluting or high on energy demand (Lei et al., 2007; Sharma et al., 2020). This necessitates the eco-friendly synthesis of WO_3_ nanoparticles. Green synthesis of never reported tungsten oxide nanoparticles was successfully achieved using the bacterium, *Stenotrophomonas maltophilia*. However, the uniformity and reproducibility of the size of nanoparticles synthesized by biological systems reduces its preference in considering various applications. In the current study a model to attain optimal combinatorial process parameter levels was successfully developed for achieving uniform sized nanoparticles. The chemical nature of the synthesized nanoparticles was confirmed by XRD and FT-IR, while its spherical morphology and size were revealed by SEM analysis. Biological systems are sensitive to environmental conditions and hence are influenced by any deviation from the optimum. Taguchi OA L27 model was used to design experiments for determining the perfect combination of the incubation parameters for *S. maltophilia*. The results obtained were analysed using statistical tools such as ANOVA and regression analysis to determine the optimal combination of the incubation parameters. Both the methods suggested ***p3t3r3d3c1*** as the best combination of the incubation parameters for achieving small sized WO_3_ nanoparticle with lowest PDI. pH of media and rate of aeration were the most influential parameters contributing 55.25% and 32.13% respectively towards the particle size of the nanoparticles and 31.62% and 47.26% respectively towards the PDI. Alteration in pH results in a change in charge and thereby the zeta potential of the metal nanoparticle. Marsalek (2014) found least stability of the ZnO nanoaprticles around their isoelectric pH, thereby influencing the size and zeta potential. Studies on synthesis of tellurium nanoparticles by Castro et al., 2020 have demonstrated a reduction in size with increased aeration rate. Temperature and time of incubation were the least influencing parameters for both particle size and PDI with negligible contribution. Contour plots developed also confirmed the same finding where different levels for temperature and time demonstrated insignificant influence and hence a lower level of these parameters may be considered to reduce the economics of the process.

Confirmatory tests performed after determining the optimal parameters for size and PDI revealed an increased corelation between predicted and experimental results. Prediction by Taguchi and regression model significantly matched with the actual values obtained for particle size and PDI of the biologically synthesized nanoparticles using the incubation parameter combination ***p3t3r3d3c1.*** The significant match among the two predictive values and the actual experimental size and PDI demonstrate the reliability of the optimization method. Linear regression analysis demonstrated a good relationship with high correlation coefficient of *R*^*2*^ *= 95.50%* for particle size and *R*^*2*^ *= 97.01%* for PDI between the measured and predicted values of particle size and PDI. The predicted equations were validated by SEM and particle size analysis, which confirmed the uniform distribution and size of the synthesized nanoparticles ranging between 70–80 nm. The application of the Taguchi method for optimizing the bacterial synthesis of tungsten oxide nanoparticles was effective and hence may be applied to other biological systems as well as chemical or industrial processes.

Bacterial synthesis of metal nanoparticles is a result of microbial evolution as a defence mechanism to vanquish the toxicity of metal ions by reducing them to less toxic or nontoxic metal oxide nanoparticles (Niño-Martínez et al., 2019). The present study reveals the tuneability of size and heterogeneity of nanoparticle production by altering the physicochemical conditions of bacterial growth.

## Declarations

### Author contribution statement

Dali Vilma Francis: Conceived and designed the experiments; Performed the experiments; Analyzed and interpreted the data; Wrote the paper.

Aiswarya T.: Analyzed and interpreted the data; Contributed reagents, materials, analysis tools or data; Wrote the paper.

Trupti Gokhale: Conceived and designed the experiments; Analyzed and interpreted the data; Contributed reagents, materials, analysis tools or data; Wrote the paper.

### Funding statement

This work was supported by BITS Pilani Dubai Campus (D1164).

### Data availability statement

No data was used for the research described in the article.

### Declaration of interest’s statement

The authors declare no conflict of interest.

### Additional information

No additional information is available for this paper.
